# The United States Chiropractic Workforce: An alternative or complement to primary care?

**DOI:** 10.1186/2045-709X-20-35

**Published:** 2012-11-21

**Authors:** Matthew A Davis, Todd A Mackenzie, Ian D Coulter, James M Whedon, William B Weeks

**Affiliations:** 1 Center for Health Policy Research, The Dartmouth Institute for Health Policy & Clinical Practice, 35 Centerra Parkway, Lebanon, NH, 03766, USA; 2 The Dartmouth Institute for Health Policy & Clinical Practice, 35 Centerra Parkway, Lebanon, NH, 03766, USA; 3 University of California at Los Angeles, CA, Senior Health Policy Analyst, RAND Corporation, Santa Monica, CA, 90401, USA; 4 Geisel School of Medicine at Dartmouth, Section of Clinical Research, HB 7505, Dartmouth Hitchcock Medical Center, One Medical Center Drive, Lebanon, NH, 03766, USA

**Keywords:** Chiropractic, Supply, Distribution, Health resources, Manpower

## Abstract

**Background:**

In the United States (US) a shortage of primary care physicians has become evident. Other health care providers such as chiropractors might help address some of the nation’s primary care needs simply by being located in areas of lesser primary care resources. Therefore, the purpose of this study was to examine the distribution of the chiropractic workforce across the country and compare it to that of primary care physicians.

**Methods:**

We used nationally representative data to estimate the per 100,000 capita supply of chiropractors and primary care physicians according to the 306 predefined Hospital Referral Regions. Multiple variable Poisson regression was used to examine the influence of population characteristics on the supply of both practitioner-types.

**Results:**

According to these data, there are 74,623 US chiropractors and the per capita supply of chiropractors varies more than 10-fold across the nation. Chiropractors practice in areas with greater supply of primary care physicians (Pearson’s correlation 0.17, p-value < 0.001) and appear to be more responsive to market conditions (i.e. more heavily influenced by population characteristics) in regards to practice location than primary care physicians.

**Conclusion:**

These findings suggest that chiropractors practice in areas of greater primary care physician supply. Therefore chiropractors may be functioning in more complementary roles to primary care as opposed to an alternative point of access.

## Background

In the United States (US) health care policymakers continue to be concerned about the ability of the primary care workforce to meet the future primary care needs of an aging and expanding population. Some authorities encourage increasing the general physician workforce to address the shortage [1-3]. However, others argue that, because of market forces, expansion would only perpetuate the overabundance of specialists and continue to widen the divide between high and low supply of primary care physicians [4,5]. Indeed, recent studies suggest that, after accounting for population differences, a 2- to 3-fold variation in physician supply exists across geographic regions [4]. And only primary care physician supply [4] appears to be related to improvements in population health outcomes [6].

To meet the nation’s future primary care needs, it might be reasonable to expand the scope of practice of ancillary practitioners such as nurse practitioners and physician assistants. However, there is concern that the number of new graduates from nurse practitioner and physician assistant programs is not keeping pace with the required needs to mitigate future primary care physician shortages [7]. In addition, there is evidence that physician assistants are providing more specialty care [8,9].

Many Americans seek care for common health conditions outside the offices of primary care physicians [10,11]. More specifically, neck and lower back conditions are among the most common conditions encountered in the primary care setting [12-15]. Chiropractors in the US provide a substantial portion of satisfactory care for these complaints [16]. In this regard, chiropractors may already be offsetting primary care shortfalls (serving as an alternative) by merely being located in areas of lesser primary care supply. If chiropractors are indeed located in areas of lesser primary care physician supply, they might be serving as points of primary contact for patients, thereby improving access. On the other hand if chiropractors are located in areas of adequate primary care physicians supply, it might suggest chiropractors function in more complementary roles (i.e. treating cases that would otherwise have been treated by primary care physicians as opposed to serving as a point of primary contact). Depending on their existing role in the larger health care system, chiropractors may be positioned to play an even larger part in addressing the nation’s growing health care needs.

However, little is actually known about the relationship between the chiropractic and primary care physician workforces [17-19]. We hypothesized chiropractors might be distributed across the nation in a manner, either directly or inversely, related to that of the nation’s primary care physician workforce. Therefore, the purpose of our study was to examine the distribution of the chiropractic workforce and compare it to that of primary care physicians.

## Methods

To examine the chiropractic workforce and compare it to that of primary care physicians, we used data from the Center for Medicare and Medicaid Service’s Unique Physician Identification Number Directory, the US Census Bureau, and data on medical physician supply previously collected by the *Dartmouth Atlas of Health Care* for 2006. The *Dartmouth Atlas of Health* Care provides national information on the distribution and use of medical services across the US according to predefined geographic units.

We gathered practitioner supply and sociodemographic population data by Zone Improvement Plan (ZIP) code and aggregated all measures according to the 306 Hospital Referral Regions (HRRs) commonly used by the *Dartmouth Atlas of Health Care*. HRRs are geographic units that were initially designed based on health care utilization and have the advantage of being independent of political boundaries.

### Measures

#### The chiropractic workforce

Estimating the national supply of chiropractors is a challenge because they can have multiple state licenses. The Unique Physician Identification Number Directory contains information on and provides a unique identification number for physicians, osteopathic physicians, and other select health care practitioners (including chiropractors) who are enrolled in the US Medicare program. Using this directory, for the years 2002 to 2008, we identified chiropractors enrolled in the Medicare program and the ZIP codes for their practice locations. In the US it is mandated that, whether they participate in billing the Centers for Medicare and Medicaid or not, chiropractors must be enrolled in Medicare in order to treat any patient over the age of 65 years. Therefore, Medicare data is likely a very complete data source for US chiropractors; albeit, it may not include the few chiropractors who, for instance, only treat children.

To confirm findings from the Medicare files, we collected data on the total number of chiropractic licenses from the Federation of Chiropractic Licensing Boards and obtained information on the total number of employed chiropractors from the National Employment Matrix (Additional file 1: Appendix 1) [20]. The National Employment Matrix is compiled biennially as part of the Employment Projections program by the US Bureau of Labor Statistics; data for the matrix comes from multiple Bureau of Labor Statistics databases including: the Occupational Employment Statistics survey (data identifying employed workforce patterns and salaries), the Current Employment Statistics Program (data on total wage in each industry), and the Current Population Survey (data on self-employed and second jobs). The dataset provides annual estimates of numbers of employed workers in specific jobs.

#### The primary care physician workforce

Data on the supply and practice location according to ZIP codes for medical physicians were used from the American Medical Association and American Osteopathic Association Masterfiles for 2006. These data were aggregated previously by the *Dartmouth Atlas of Health Care* and excluded physicians in graduate medical education, those working primarily in teaching, administrative, or research positions and those working less than 20 hours per week in clinical settings. The Masterfiles contain information on 406 self-designated specialties by physicians.

The Masterfiles were used to determine the relative overlap in the local supply of chiropractors and primary care physicians, operationalized as physicians who self-reported practicing internal medicine, family medicine, or general practice (note: we did not include pediatricians in our definition).

#### Population data

Using extrapolated 2000 US Census Bureau data we generated estimates of the number of chiropractors and primary care physicians per 100,000 adults (age ≥ 18 years) for 2006. We limited our analysis to the adult population age 18 and older because that population uses approximately 80 to 90 percent of all chiropractic services each year [16]. Thus we were consistent with the potential treatment population in our definition of primary care physicians. For each Dartmouth Atlas defined HRR, [21] we generated the supply of practitioners per 100,000 adult capita according to the US Census Bureau 2006 population data. We also obtained other sociodemographic data for HRRs, including age, race/ethnicity, family income, and education from the US Census Bureau.

To examine population factors that might influence the supply of practitioners, we calculated the percent of the adult population aged 65 and older, those aged 25 or older with a high school education or less, those of racial or ethnic minority, and families with a median household income < $20,000 for each HRR.

Data from Washington University’s Rural Health Research Center were used to classify US ZIP codes as either urban or rural [22]. The Rural Health Research Center uses population data from the US Census Bureau as well as spatial data and commuting distances to generate rural classification scores known as Rural–urban Commuting Area codes. Rural–urban Commuting Area codes were collapsed into either urban or rural and determined the percent of the adult population that resided in rural zip codes for each HRR.

### Statistical analyses

We used Pearson’s correlation to compare the relationship between per adult capita supply of chiropractors to that of primary care physicians and determined the expected supply of chiropractors per HRR when accounting for population size and age. We operationally defined very high and very low chiropractic supply areas as HRRs wherein observed supply was at least 50 percent higher or lower than expected supply, respectively (observed to expected ratio of 1.5 for “Very High” and 0.5 for “Very Low”). To examine the relationship between population characteristics and the supply of the chiropractors and primary care physicians we used multiple variable Poisson regression adjusted for population size. Sandwich variance was used to generate more robust standard error estimates. We used ArcGIS version 10.0 (ESRI, Redlands, CA) for spatial analyses and Stata version 11.0 (College Station, Texas) for regression analyses.

## Results

We estimated that there were 74,623 chiropractors in the US who held 87,237 state chiropractic licenses (a mean of 1.2 licenses per practitioner) in 2006. The national chiropractic workforce has been relatively stable from 2002 to 2008 (Additional file 1: Appendix 1). There were about twice as many active primary care physicians (169,843) in 2006.

**Figure 1:**
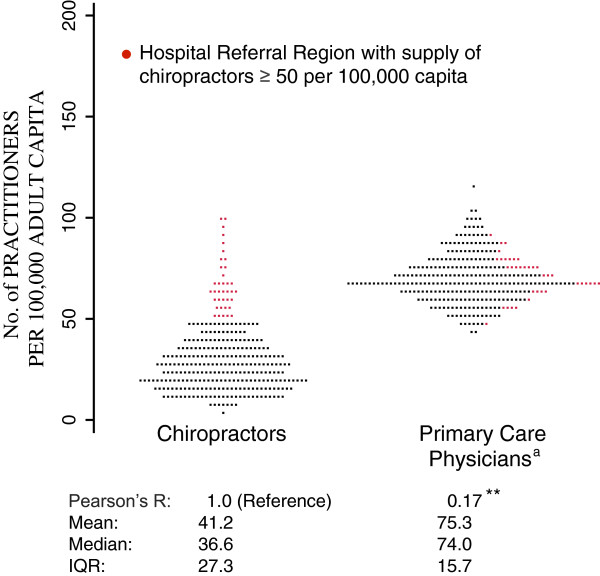
**Distribution of Practitioners per United States Hospital Referral Region.** Abbreviations: IQR, interquartile range. a: Primary care physicians defined as general practitioners, internal medicine, and family medicine physicians (pediatricians excluded). ** p-value < 0.01.

The per capita supply of chiropractors ranged from 10.7 per 100,000 capita in Bryan, Texas to 126.4 per 100,000 capita in Mason City, IA, a 10.8-fold variation in supply. Although New Orleans had an even lower supply of chiropractors (6.8 per 100,000 capita) than Bryan, because the low numbers in New Orleans could have been an effect of chiropractors relocating after Hurricane Katrina in 2005, we were concerned that the New Orleans results were spurious and eliminated New Orleans from further analysis. The median supply of chiropractors was 36.6 per 100,000 capita. The supply of primary care physicians ranged from 46.0 to 123.8 per 100,000, with a median supply of 74.0 per 100,000, a considerably lower 1.7-fold variation across HRRs (Figure 1).

The supply of chiropractors and primary care physicians was correlated (Pearson correlation coefficient = 0.17, p-value < 0.01). However, the 20% of HRRs with a high supply of chiropractors per capita (defined as those greater than or equal to 50 per 100,000 capita) were roughly equally distributed across high and low primary care physician supply locales (Figure 1).

**Figure 2:**
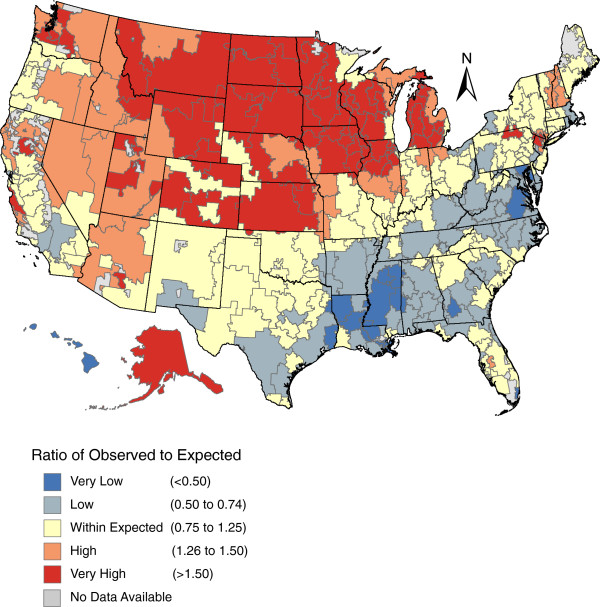
**The Ratio of Observed to Expected^a^ Supply of Chiropractors per United States Hospital Referral Region.** a: Expected counts generated from Poisson regression model adjusted for total population and age.

We found that variation in the supply of chiropractors across the US exhibited a specific spatial pattern (Figure 2). The Midwestern and Western regions contained considerably more chiropractors than expected after accounting for differences in population age and size — a similar but more pronounced pattern than that of the crude supply of chiropractors per 100,000 capita (Additional file 1: Appendix 2). Conversely, the Southern portion of the US had considerably fewer chiropractors than expected. Considering the high supply of medical physicians in the Northeast, we were surprised to find that few HRRs in the Northeast exceeded expected supply of chiropractors. New Hampshire and Vermont Hospital Referral Areas were an exception and some of these HRRs trended towards higher concentrations of chiropractors (a ratio of 1.26 to 1.50 of observed to expected).

### Population characteristics and practitioner supply

**Figure 3:**
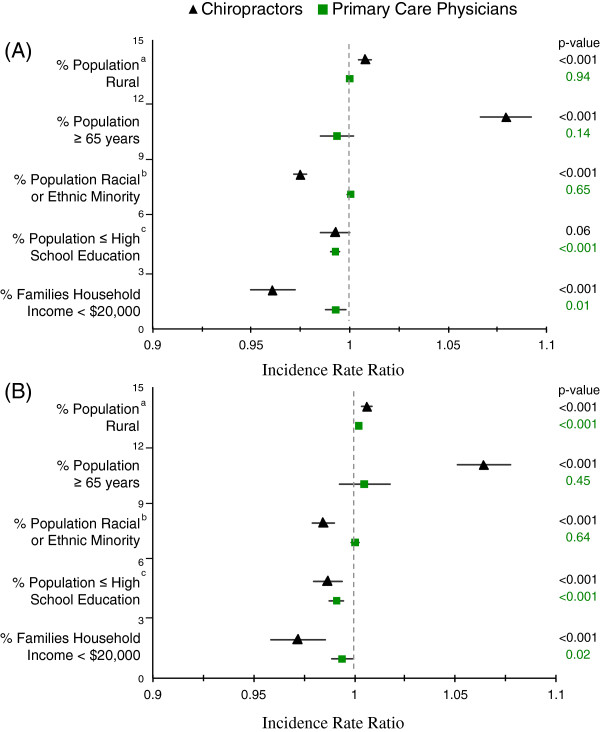
**(A) Results of Univariate and (B) Multiple Variable Poisson Regression Models for the Association between Practitioner Supply and Population Characteristics.** a: Based on urban versus rural as defined by University of Washington’s Rural–urban Commuting Area Codes. b: Minority races and ethnicities include all adults that are not Non-Hispanic White. c: Among adults ≥ 25 years old (all other measures among adults ≥ 18 years).

In both univariate and multiple variable Poisson regression models, population characteristics were more strongly associated with the supply of chiropractors than with that of primary care physicians (Figure 3). Population, age, and rurality were associated with higher supply of chiropractors. For instance, every 1% increase in the 65 and older population was associated with a 6.4% increase in the supply of chiropractors. Additionally, a lower proportion of minorities, those with greater than high school education, and those with income greater than $20,000 were predictive of a higher supply of chiropractors (p-value ≤ 0.001 for all three measures) (Figure 3B).

Lower population educational levels were predictive of lower chiropractor and primary care physician supply, p-value ≤ 0.001. A higher proportion of the population living in rural settings was predictive of higher supply of chiropractors in both univariate and multiple variable models (p-value < 0.001 for both) and primary care physicians in the multiple variable model.

## Discussion

This is one of the few recent studies to examine the national supply of chiropractors [17,23-25] and, to our knowledge, the first to compare regional variation in the supply of chiropractors to that of primary care physicians. We estimate the total chiropractic workforce to be over 74,000 practitioners, almost half of our estimate of 169,843 *active* primary care physicians in 2006. We found greater than a 10-fold variation across the country in the supply of chiropractors, a higher concentration of chiropractic services than expected in the Midwest, and a lower one in the South which collaborates with a recent report that examined the influence of supply on utilization [25]. Finally, we found a statistically significant correlation with the per capita supply of primary care physicians.

Our study suggests that chiropractors tend to establish their practices in areas that also have higher supply of primary care physicians. This is likely explained by the influence of market conditions on both groups: both are likely to locate in areas that will support their practices – however, there are many other personal and professional factors that our data did not account for. This finding may suggests that chiropractors function in a complementary role, as opposed to an alternative role, to primary care physicians. Whether that complementary role is substitutive or additive is unknown.

When chiropractors and physicians are asked independently to define the role primary care plays in population health there is significant agreement between the two professions [26]. However, chiropractors in the US remain largely considered to be neuromuscular specialists. In addition to government and private insurances designating chiropractors' as specialists in health care systems, chiropractors role as neuromuscular specialists is also is due to patients’ impressions of their services – patients tend to view chiropractors as “back doctors” not as primary care providers [27]. Many within the US chiropractic profession are content with their role as neuromuscular specialists and those who wish to provide more primary care roles likely have numerous barriers to overcome [28].

Previous studies have examined the professional relationship between primary care physicians and chiropractors and uncovered an overall lack of inter-professional cooperation [29-31]. There is considerable overlap in the patient population treated by primary care physicians and chiropractors - neck and back conditions are among the most common complaints in the primary care setting [15]. This, coupled with our finding that chiropractors tend to locate in areas with higher primary care physician supply, suggests there is considerable potential for inter-professional collaboration. Health service use for neck and back conditions has risen in recent years and are attributed with substantial costs to the US economy [12,13,32]. Novel, interdisciplinary strategies are needed to address the growing needs of this population. Improved collaboration and coordinating care between chiropractors and primary care physicians has potential to help improve population health and reduce health care costs.

The relatively strong influence of population characteristics on the regional supply, of chiropractic care suggests that chiropractors are more responsive to market conditions than primary care physicians. Indeed, the chiropractic profession in the US continues to be predominately a “cottage industry”, and therefore most chiropractors are also small business owners. It is likely that chiropractors establish their practices in areas of higher socioeconomic status, health care access, and overall demand for their services.

Also, there appears to be a relationship between rurality and chiropractic supply, and previous reports have explored the relationship [17,24,33]. For instance, one study found that chiropractors practicing in a Health Professional Shortage Area (which tend to be more rural) had larger patient panels than those not in Health Professional Shortage Areas, suggesting chiropractors may be playing a role in improving access to health services in certain locales [24]. Our results align with most previous reports as we found more rural areas to be predictive of a higher supply of chiropractors. Nevertheless, while chiropractors tend to be located in more rural areas (which may improve access to care in some populations), they are less likely to be located in poorer areas and areas with high proportions of racial and ethnic minorities.

These findings will inform health care policymakers as they decide how chiropractors will be part of US health care reform efforts, such as Accountable Care Organizations [34] or the potential expansion of coverage of chiropractic services. The size of the chiropractic workforce is relatively large, with higher concentrations in the Midwest, a pattern not exhibited among medical physician groups [21]. The extent to which chiropractors might be able to supplement medical care should be considered as a way to enhance the primary care workforce. Specifically, even though chiropractors might not be serving as points of primary contact role, chiropractors may be contributing to addressing the continued health care related to back and neck conditions particularly for populations residing in rural locales.

### Limitations of the study

Our study has several limitations. First, we examined chiropractors who were enrolled in Medicare with the Centers for Medicare and Medicaid Services. Using data from the Centers for Medicare and Medicaid Services to estimate the national chiropractic workforce may have either underestimated the national supply (by excluding the few chiropractors not enrolled with Medicare) or overestimated it by including chiropractors working less than full-time. Nevertheless, our data regarding the chiropractic workforce appeared consistent based on other sources (Additional file 1: Appendix 1). Additionally, we used previously collected data that sought to make conservative estimates of the US primary care workforce by excluding physicians working less than 20 hours per week and those in primarily academic positions. Therefore potential underestimation of the primary care physician workforce may have affected our comparisons of the overall estimates of the two workforces exaggerating the size of the chiropractic workforce when compared to that of primary care physicians.

We used geographic units (the 306 HRRs), which were defined by regional use of specialty medical services. It is unclear whether medical services utilization patterns are comparable to those for ancillary health services such as chiropractic care. Given the size of the chiropractic workforce, HRRs are currently the only feasible geographic unit for study and does have the advantage of being independent of political borders. Lastly, because our study consisted of analyses on the ecological level, which rely on aggregate statistics and are inherently descriptive, our findings do not necessarily represent associations at the level of the individual practitioner. Despite the inherent limitations of our study design, this study provides important information regarding US chiropractic workforce and its relationship to that of the primary care physicians.

## Conclusions

As the US health care system begins to recognize and reimburse more for preventive interventions, services such as chiropractic care may find more opportunities for integration with primary care. On a per visit basis, chiropractic care is inexpensive when compared to medical care [35] but larger studies are required to more thoroughly evaluate the costs associated with specific episodes of care as well as any potential indirect effects on overall cost. In light of increasing demands on an already overextended primary care workforce, it is an important time for health care policymakers to consider the direct and indirect effects of decisions regarding future coverage of chiropractic care [34]. Health care policymakers and other stakeholders should consider these findings when planning for the future health care needs of the nation.

## Competing interests

The authors declare that they have no competing interests.

## Authors' contributions

*Study concept and design:* MAD and WBW. *Analysis and interpretation of data:* MAD, IDC, and TAM. *Critical revision of the manuscript for important intellectual content:* IDC, JMW, and WBW. *Statistical analysis:* MAD and TAM. *Administrative, technical, or material support:* MAD. *Study supervision:* MAD and WBW. All authors read and approved the final manuscript.

### Disclaimer

The views expressed herein do not necessarily represent the official views of the National Center for Complementary & Alternative Medicine or the National Institutes of Health.

### Financial disclosures and funding/support

Davis was supported by Award Number K01AT006162 from the National Center for Complementary & Alternative Medicine and Whedon was supported by Award Number K01AT005092 from the National Center for Complementary & Alternative Medicine.

## Supplementary Material

Additional file 1 **Appendix 1.** The National Supply of Chiropractors from Three Different Sources, 2002 to 2008. a: No. of State Chiropractic Licenses, Federation of Chiropractic Licensing Boards. b: No. of Chiropractors enrolled with Center for Medicare & Medicaid Services. c: No. of Chiropractors reported in the United States Bureau of Labor Statistic’s Employment Matrix. Note: In 2007 and 2008 Medicare initiated replacement of the Unique Physician Identifier with the National Provider Identifier which may explain the decline in estimates. **Appendix 2** Supply of Chiropractors per United States Hospital Referral Region. Click here for file

## References

[B1] Cooper RA Weighing the evidence for expanding physician supply Ann Intern Med 2004 141 705 714 15520427 10.7326/0003-4819-141-9-200411020-00012

[B2] Cooper RA Myth and reality underlying the needed expansion of graduate medical education Gastroenterology 2009 136 2045 2047 10.1053/j.gastro.2009.04.024 19406135

[B3] Cooper RA Expanding physician supply - an imperative for health care reform Pharos Alpha Omega Alpha Honor Med Soc 2010 73 35 37 20458788

[B4] Goodman DC Grumbach K Does having more physicians lead to better health system performance? JAMA 2008 299 335 337 10.1001/jama.299.3.335 18212319

[B5] Goodman DC Twenty-year trends in regional variations in the U.S. physician workforce 2004 Suppl Variation: Health Aff (Millwood) 90 97 10.1377/hlthaff.var.90 15471767

[B6] Chang CH Stukel TA Flood AB Goodman DC Primary care physician workforce and Medicare beneficiaries' health outcomes JAMA 2011 305 2096 2104 10.1001/jama.2011.665 21610242 PMC3108147

[B7] Cooper RA New directions for nurse practitioners and physician assistants in the era of physician shortages Acad Med 2007 82 827 828 10.1097/ACM.0b013e31812f7939 17726384

[B8] Jones PE Physician assistant education in the United States Acad Med 2007 82 882 887 10.1097/ACM.0b013e31812f7c0c 17726400

[B9] Jones PE Cawley JF Workweek restrictions and specialty-trained physician assistants: potential opportunities J Surg Educ 2009 66 152 157 10.1016/j.jsurg.2009.03.033 19712914

[B10] Barnes PM Bloom B Nahin RL Complementary and alternative medicine use among adults and children: United States, 2007 National Health Statistics Reports 2008 12 1 23 19361005

[B11] Davis MA West AN Weeks WB Sirovich BE Health behaviors and utilization among users of complementary and alternative medicine for treatment versus health promotion Health Serv Res 2011 46 1402 1416 10.1111/j.1475-6773.2011.01270.x 21554272 PMC3207184

[B12] Martin BI Deyo RA Mirza SK Turner JA Comstock BA Hollingworth W Sullivan SD Expenditures and health status among adults with back and neck problems JAMA 2008 299 656 664 10.1001/jama.299.6.656 18270354

[B13] Martin BI Turner JA Mirza SK Lee MJ Comstock BA Deyo RA Trends in Health Care Expenditures, Utilization, and Health Status Among US Adults With Spine Problems, 1997-2006 Spine (Phila Pa 1976) 2009 34 2077 2084 10.1097/BRS.0b013e3181b1fad1 19675510

[B14] Andersson GB Epidemiological features of chronic low-back pain Lancet 1999 354 581 585 10.1016/S0140-6736(99)01312-4 10470716

[B15] Hart LG Deyo RA Cherkin DC Physician office visits for low back pain. Frequency, clinical evaluation, and treatment patterns from a U.S. national survey Spine (Phila Pa 1976) 1995 20 11 19 10.1097/00007632-199501000-00003 7709270

[B16] Cherkin DC Deyo RA Sherman KJ Hart LG Street JH Hrbek A Davis RB Cramer E Milliman B Booker J Mootz R Barassi J Kahn JR Kaptchuk TJ Eisenberg DM Characteristics of visits to licensed acupuncturists, chiropractors, massage therapists, and naturopathic physicians J of Am Board Fam Med 2002 15 463 472 12463292

[B17] Smith M Carber LA Chiropractors as safety net providers: first report of findings and methods from a US survey of chiropractors J Manipulative Physiol Ther 2007 30 718 728 10.1016/j.jmpt.2007.11.001 18082744

[B18] Smith M Morschhauser S Establishing a database of U.S. chiropractic health manpower data: furthering the development of research infrastructure 1999 Meeting: Association for Health Services Research

[B19] Davis MA Davis AM Luan J Weeks WB The supply and demand of chiropractors in the United States from 1996 to 2005 Altern Ther Health Med 2009 15 36 40 19472863

[B20] United States Bureau of Labor Statistics Employment Projections: National Employment Matrix: 29-1011 Chiropractors 2011 Available at: http://www.bls.gov/emp/empoils.htm

[B21] Goodman DC Fisher ES Bronner KK Hospital and Physician Capacity Update: A Brief Report from the Dartmouth Atlas of Health Care 2009 Lebanon, NH: The Dartmouth Institute for Health Policy and Clinical Practice 36375004

[B22] RUCA Rural Health Research Center 2011 http://depts.washington.edu/uwruca/

[B23] Cooper RA McKee HJ Chiropractic in the United States: trends and issues Milbank Q 2003 81 107 138 10.1111/1468-0009.00040 12669653 PMC2690192

[B24] Smith M Carber L Chiropractic health care in health professional shortage areas in the United States Am J Public Health 2002 92 2001 2009 10.2105/AJPH.92.12.2001 12453823 PMC1447366

[B25] Whedon JM Song Y Davis MA Lurie JD Use of Chiropractic Spinal Manipulation in Older Adults is Strongly Correlated with Supply Spine (Phila Pa 1976) 2012 37 1771 7 10.1097/BRS.0b013e31825762b7 22487711 PMC3414681

[B26] Gaumer GL Walker A Su S Chiropractic and a new taxonomy of primary care activities J Manipulative Physiol Ther 2001 24 239 259 10.1067/mmt.2001.114366 11353936

[B27] Teitelbaum M The role of chiropractic in primary care: findings of four community studies J Manipulative Physiol Ther 2000 23 601 609 10.1067/mmt.2000.110945 11145800

[B28] Gaumer G Koren A Gemmen E Barriers to expanding primary care roles for chiropractors: The role of chiropractic as primary care gatekeeper J Manipulative Physiol Ther 2002 25 427 449 10.1067/mmt.2002.126474 12214185

[B29] Greene BR Smith M Allareddy V Haas M Referral patterns and attitudes of primary care physicians towards chiropractors BMC Complement Altern Med 2006 6 5 10.1186/1472-6882-6-5 16509963 PMC1456998

[B30] Allareddy V Greene BR Smith M Haas M Liao J Facilitators and barriers to improving interprofessional referral relationships between primary care physicians and chiropractors J Ambul Care Manage 2007 30 347 354 17873667 10.1097/01.JAC.0000290404.96907.e3

[B31] Curtis P Bove G Family physicians, chiropractors, and back pain J Fam Pract 1992 35 551 555 1431771

[B32] Davis MA Onega T Weeks W Lurie J Where the United States Spends its Spine Dollars: Expenditures on different ambulatory services for the management of back and neck conditions Spine (Phila Pa 1976) 2012 37 1693 1701 10.1097/BRS.0b013e3182541f45 22433497 PMC3423501

[B33] Cote P Cassidy JD Carroll L The treatment of neck and low back pain: who seeks care? who goes where? Med Care 2001 39 956 967 10.1097/00005650-200109000-00006 11502953

[B34] Davis MA Whedon JM Weeks WB Complementary and alternative medicine practitioners and Accountable Care Organizations: the train is leaving the station J Altern Complement Med 2011 17 669 674 10.1089/acm.2011.0364 21732823 PMC3142629

[B35] Davis MA Sirovich BE Weeks WB Utilization and expenditures on chiropractic care in the United States from 1997 to 2006 Health Serv Res 2010 45 748 761 20002763 10.1111/j.1475-6773.2009.01067.x PMC2875758

