# Supplementary material for: The United States Chiropractic Workforce: An alternative or complement to primary care?
Source: Chiropr Man Therap. 2012 Nov 21;20:35. doi: 10.1186/2045-709X-20-35 (PMC3551710; doi:10.1186/2045-709X-20-35)
Supplement: Additional file 1 — Appendix 1. The National Supply of Chiropractors from Three Different Sources, 2002 to 2008. a: No. of State Chiropractic Licenses, Federation of Chiropractic Licensing Boards. b: No. of Chiropractors enrolled with Center for Medicare & Medicaid Services. c: No. of Chiropractors reported in the United States Bureau of Labor Statistic’s Employment Matrix. Note: In 2007 and 2008 Medicare initiated replacement of the Unique Physician Identifier with the National Provider Identifier which may explain the decline in estimates. Appendix 2 Supply of Chiropractors per United States Hospital Referral Region. [file 2045-709X-20-35-S1.pdf]

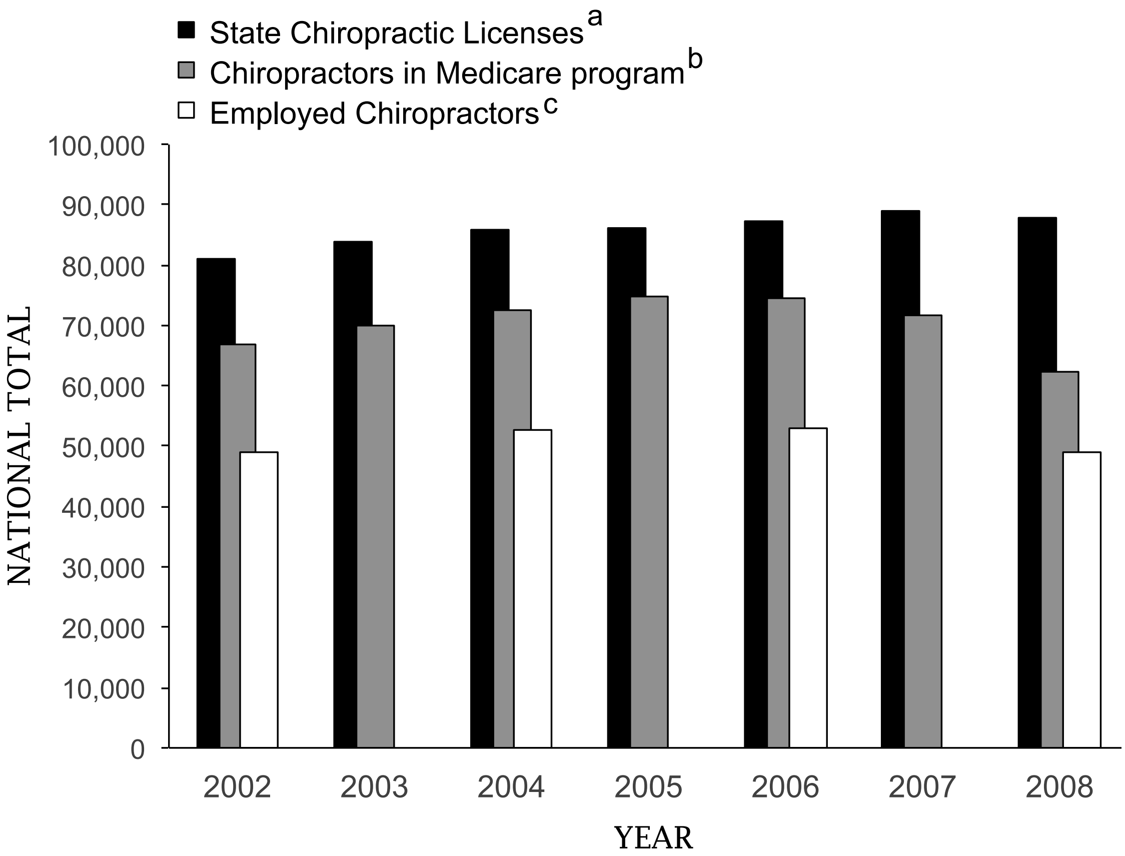

Appendix 1. The National Supply of Chiropractors from Three Different Sources, 2002 to 2008.

a: No. of State Chiropractic Licenses, Federation of Chiropractic Licensing Boards
b: No. of Chiropractors enrolled with Center for Medicare & Medicaid Services
c: No. of Chiropractors reported in the United States Bureau of Labor Statistic's Employment Matrix
Note: In 2007 and 2008 Medicare initiated replacement of the Unique Physician Identifier with the National Provider Identifier which may explain the decline in estimates.

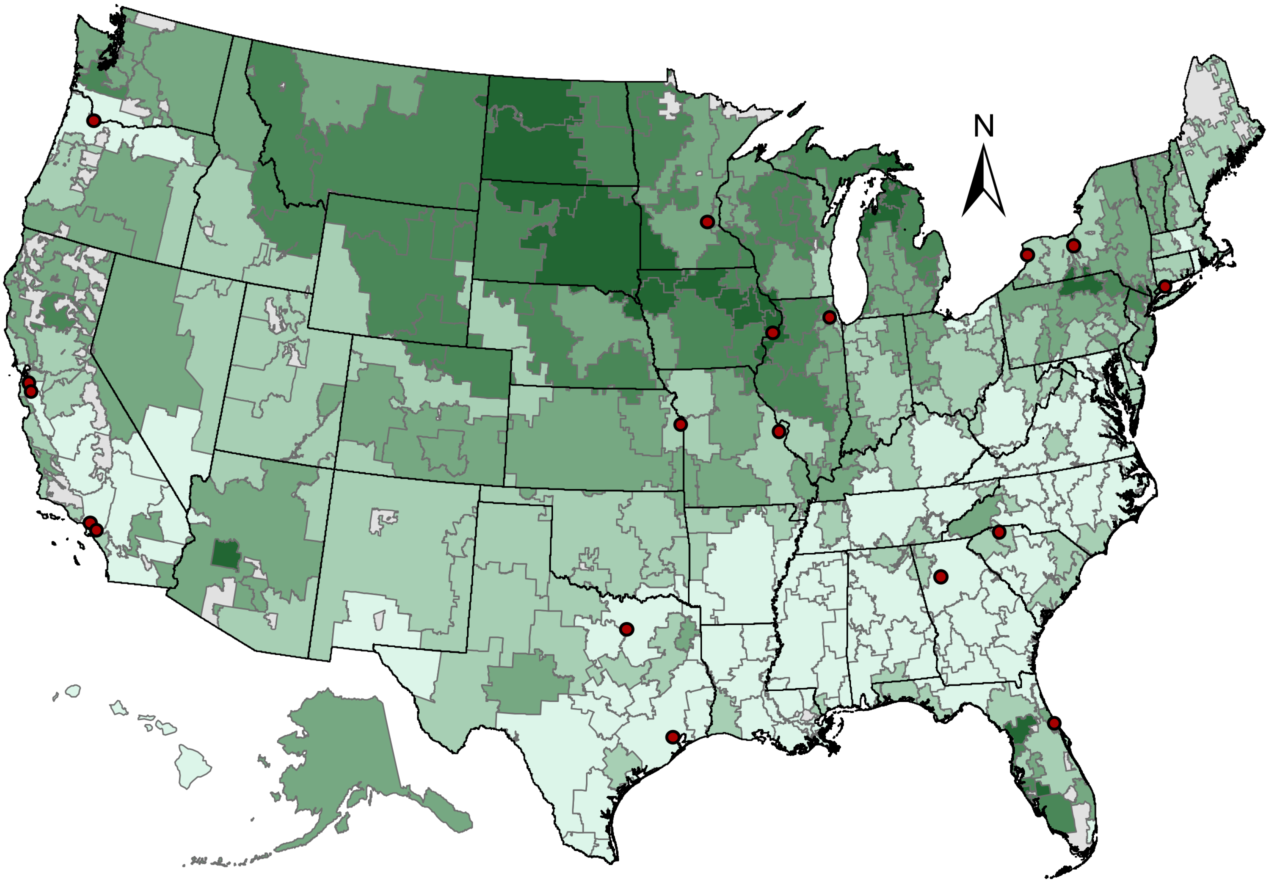

Chiropractors per 100,000 Adult Capita

- Quintile 1 (6.9 - 25.5) - Lowest
- Quintile 2 (25.6 - 40.0)
- Quintile 3 (39.9 - 57.5)
- Quintile 4 (57.6 - 84.9)
- Quintile 5 (85.0 - 126.4) - Highest
- No Data Available
- Chiropractic Schools

Appendix 2. Supply of Chiropractors per United States Hospital Referral Region.
